# Biomarkers in head and neck squamous cell carcinoma: unraveling the path to precision immunotherapy

**DOI:** 10.3389/fonc.2024.1473706

**Published:** 2024-10-08

**Authors:** Kamal S. Saini, Sasikala Somara, Heidi C. Ko, Purva Thatai, Angela Quintana, Zachary D. Wallen, Michelle F. Green, Ravi Mehrotra, Sandra McGuigan, Lingjuan Pang, Soma Das, Kavita Yadav, Dobrica Neric, Luca Cantini, Chinmayee Joshi, Kazuya Iwamoto, Sudha Dubbewar, Laura Vidal, Isagani Chico, Eric Severson, Luigi Lorini, Sunil Badve, Paolo Bossi

**Affiliations:** ^1^ Fortrea Inc., Durham, NC, United States; ^2^ Addenbrooke’s Hospital, Cambridge University Hospitals NHS Foundation Trust, Cambridge, United Kingdom; ^3^ Labcorp Oncology, Durham, NC, United States; ^4^ Vall d’Hebron Institute of Oncology, Barcelona, Spain; ^5^ Emory University, Atlanta, GA, United States; ^6^ George Institute for Global Health, New Delhi, India; ^7^ Medical Oncology and Hematology Unit, IRCCS Humanitas Cancer Centre, Milan, Italy; ^8^ Università degli Studi di Brescia, Brescia, Italy

**Keywords:** head and neck cancer, predictive biomarker, prognostic biomarker, diagnostic biomarker, squamous cell cancer

## Abstract

Recent strides in understanding the molecular underpinnings of head and neck cancers have sparked considerable interest in identifying precise biomarkers that can enhance prognostication and enable personalized treatment strategies. Immunotherapy has particularly revolutionized the therapeutic landscape for head and neck squamous cell carcinoma, offering new avenues for treatment. This review comprehensively examines the application and limitations of the established and emerging/novel biomarkers for head and neck squamous cell carcinoma. Established biomarkers, including well-characterized genetic mutations, protein expressions, and clinical factors, have been extensively studied and validated in clinical practice. Novel biomarkers identified through molecular analyses, including novel genetic alterations, immune-related markers, and molecular signatures, are currently being investigated and validated in preclinical and clinical settings. Biomarkers hold the potential to deepen our understanding of head and neck squamous cell carcinoma biology and guide therapeutic strategies. The evolving paradigm of predictive biomarkers facilitates the study of individual responses to specific treatments, including targeted therapy and immunotherapy.

## 1 Introduction

Head and neck cancer accounts for approximately 5.0% of all cancer cases globally, with an estimated 947,211 new cases and 482,428 deaths (5.0% of global cancer deaths), in 2022 (1). Head and neck cancers encompass a range of etiologically and histologically diverse malignancies involving several anatomically contiguous structures, including the skin, upper aerodigestive tract, salivary glands, sinonasal tract, ear and temporal bone. Up to 90% of head and neck cancers are squamous cell carcinomas (SCC) arising from the mucosa or the skin (2). The incidence of HNSCC is on the rise in many countries around the globe, especially in younger populations, with a predicted 30% annual increase in incidence by 2030 (3, 4). Epidemiological studies by the International Agency for Research on Cancer of the World Health Organization have identified factors such as tobacco use, alcohol consumption and their combination, exposure to environmental pollutants, and viral infections like human papillomavirus (HPV) and Epstein-Barr virus (EBV) as risk factors for HNSCC (5, 6). The use of areca nut or betel quid products has also been linked with a high incidence of oral cavity cancer particularly in India, Taiwan and certain provinces in mainland China (7, 8).

The majority of patients with HNSCC are found to have a locally advanced disease and approximately 10% of these patients already have developed metastases at the time of diagnosis (9–11). Standard treatment options for HNSCC include surgery, radiotherapy, and/or chemotherapy (12, 13). Despite significant advancements in these therapies, patients with advanced-stage disease remain at a considerable risk of mortality, with 5-year relative survival rates of approximately 60% (14). Moreover, patients who experience recurrence (observed in 40%-60% within 3 years) or develop metastases tend to have a poor prognosis (13, 15).

**Figure 1:**
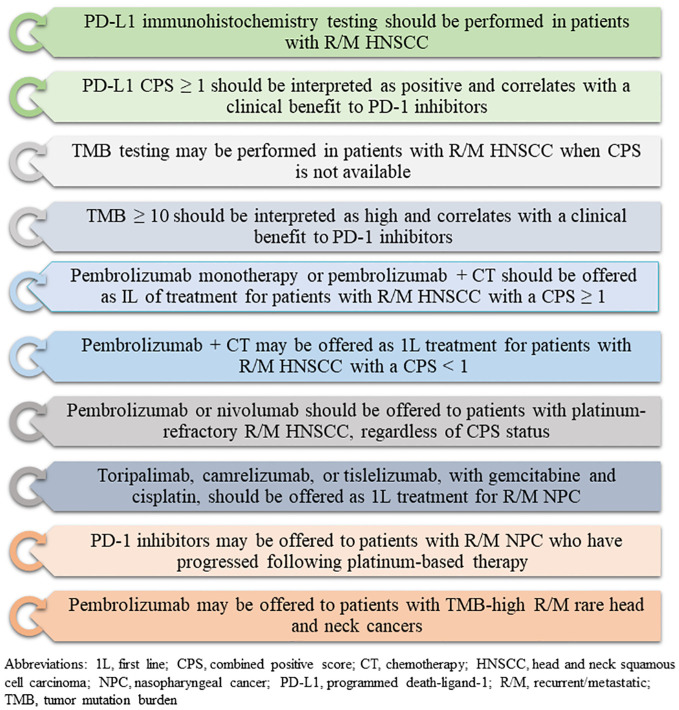
Recommendations for biomarker testing and immunotherapy for head and neck cancers (based on American Society of Clinical Oncology guidelines, 2022).

Until 2019, the combination of cetuximab, cisplatin, and 5-fluorouracil (5-FU) was the preferred initial systemic treatment for recurrent/metastatic HNSCC (16). The treatment paradigm of HNSCC changed rapidly with the emergence of immunotherapy. Currently, multiple immunotherapy strategies are being explored, including immune checkpoint inhibitors (ICIs), co-stimulatory agonists, antigenic vaccines, oncolytic virus therapy, and adoptive T-cell transfer (17). Among these, ICIs have gained particular prominence, with drugs like pembrolizumab and nivolumab emerging as preferred options for recurrent/metastatic HNSCC (18–20). However, a significant proportion of patients endure immune-related adverse effects from ICI therapy without experiencing clinical advantages as well as the primary and secondary resistance to ICI therapy represents an unmet medical need (11, 19). Therefore, to ensure optimal patient selection for immunotherapy, there is a critical need to identify reliable and practical predictive biomarkers, along with prognostic biomarkers. The American Society of Clinical Oncology has recently issued guidelines to assist health care practitioners and patients in navigating immunotherapy and biomarker testing for HNSCC (**Figure 1**) (12). Similarly, the Spanish Society of Medical Oncology and Pathology has provided a consensus outlining the most relevant predictive biomarkers for HNSCC and offering a guide for their determination and interpretation (21). The widespread adoption of biomarkers in clinical practice may ensure that patients receive the most effective and targeted treatments available, thereby maximizing therapeutic outcomes and improving overall patient care in the field of oncology. In the current paper, we present a comprehensive review of predictive biomarkers for immune checkpoint inhibition besides existing diagnostic and prognostic biomarkers of HNSCC.

## 2 Diagnostic and prognostic biomarkers in HNSCC

### 2.1 Diagnostic biomarkers in HNSCC

While biopsy remains the gold standard for diagnosis of HNSCC, its complexity and limited accessibility have prompted the exploration of alternative diagnostic approaches. Serum-based clinical testing emerges as a relatively non-invasive method for diagnosing HNSCC (22). While numerous biomarkers have been proposed to significantly impact the diagnosis and prognosis of HNSCC, only a few of them have been validated for clinical use (23). The Transparent Reporting of a multivariable prediction model for Individual Prognosis or Diagnosis (TRIPOD) initiative has recommended guidelines for the reporting studies involved in the development, validation, or updating a prediction model, whether for diagnostic, or prognostic purposes (24). **Table 1** presents a compilation of diagnostic biomarkers studied covering the different -omics fields for the diagnosis of HNSCC.

**Table 1:** Diagnostic biomarkers investigated for head and neck squamous cell carcinoma (Source: Konings et al., 2020; Front. Oncol.) (138).

ANATOMICAL TUMOR	POTENTIAL BIOMARKER	TYPE OF SAMPLE	EXPRESSION	TEST RESULTS
GENOMICS				
**(PROMOTOR) HYPERMETHYLATION**				
Oral SCC	**A promotor hypermethylation panel (HOXA9 and NID2)**	Tissue biopsies	Downregulation	Sensitivity - 94% Specificity - 75%
Oral SCC, Oropharyngeal SCC	**Methylation of cg01009664 of the thyrotropin-releasing hormone gene**	Oral rinse, and oral swab	Downregulation	Sensitivity - 91.3% (swab) and 86.15% (rinse) Specificity - 84.85% (swab) and 89.66% (rinse)
Oral SCC	**Promotor hypermethylation panel (PTEN and p16)**	Tissue biopsy	Downregulation	Not specified
**EBV-RELATED MARKERS**				
NPC	**IgA VCA + EBV DNA load**	Blood sample	Upregulation	Sensitivity - 99% Specificity - 96-98%
**HPV-RELATED MARKERS**				
Oropharyngeal carcinoma, HNSCC	**HPV-16 E16 antibodies**	Blood sample	Seropositive	Sensitivity - 96% Specificity - 98% Accuracy - 97%
**miRNA**				
Oral cancer	**Combination of miR-196a and miR-196b**	Blood sample	Upregulation	Sensitivity - 88% Specificity - 93%
Oral leukoplakia, Oral SCC	**Three-plasma miRNA panel (miR-222-3p, miR-150-5p, and miR-423-5p)**	Blood sample	Upregulation	Not specified
Larynx carcinoma	**Combination of hsa-miR-657 and hsa-miR-1287**	Tissue biopsy	Downregulation/ Upregulation	Sensitivity - 86.21% Specificity - 100%
Laryngeal SCC	**miR-155**	Tissue biopsy, blood sample	Upregulation	Sensitivity- 58.4% Specificity - 69.5%
**INTERFERONS**				
Oral SCC	**Interferon inducible transmembrane protein 1 and ISG15**	Tissue biopsy	Upregulation	Not specified
**OTHERS**				
Oral SCC	**5-hydroxylmethylcytosine**	Tissue Biopsy	Downregulation	Not specified
HNSCC, especially oropharyngeal SCC	**Total cfDNA from plasma**	Blood sample	Upregulation	Not specified
PROTEOMICS				
**CYTOKINES**				
Oral leukoplakia with dysplasia tongue SCC	**IL-6**	Saliva	Upregulation	Not specified
**OTHERS**				
Oral SCC	**RACK1**	Tissue biopsy	Upregulation	Not specified
Oral SCC and oral epithelial dysplasia lesions	**Phosphorylation of** **ribosomal protein s6 (p-RPS6)**	Tissue biopsy	Upregulation	Not specified
HNSCC	**Midkine**	Blood sample	Upregulation	Sensitivity - 57.3% Specificity - 85.3%
HNSCC	**Salivary total protein + soluble CD44 levels**	Salivary rinse	Upregulation	Sensitivity - 62-79% Specificity - 88-100%
GLYCOMICS				
Oropharyngeal carcinoma and oral cancer	**Total sialic acid/total protein ratios and α-l-fucosidase**	Blood sample, saliva	Upregulation	Sensitivity - 88.2% (serum) and 61.2% (saliva) Specificity - 57.2% (serum) and 44.3% (saliva)
Oral SCC	**Sialic acid, total protein, total sugar**	Saliva	Upregulation	Not specified
METABOLOMICS				
Oral SCC	**Altered energy metabolism**	Blood sample	Upregulation/ Downregulation	Not specified
Oral SCC	**A panel of 4 metabolites: choline, betaine, pipecolinic acid, L-carnitine**	Saliva	Upregulation/ Downregulation	Sensitivity - 100% Specificity - 96.7% Accuracy - 99.7%
Oral SCC	**Salivary glycine and proline**	Saliva	Downregulation	Not defined
RADIOMICS				
HNSCC	**MRI**	Not applicable	Not applicable	Sensitivity - 84% Specificity - 70% Accuracy - 79%

cfDNA, cell free DNA; EBV, Epstein-Barr virus; HNSCC, head and neck squamous cell carcinoma; IL, interlukin; miRNA, microRNA; MRI, magnetic resonance imaging; NPC, nasopharyngeal carcinoma; SCC, squamous cell carcinoma.

### 2.2 Prognostic biomarkers in HNSCC

Prognostic markers in HNSCC offer valuable insights into disease progression and patient outcomes. These markers, ranging from molecular signatures to clinical parameters, help clinicians to tailor treatment strategies and predict the likelihood of recurrence or metastasis, thereby improving patient management and survival rates.

#### 2.2.1 Human papillomavirus infection status

The human papillomavirus infection is a predominant cause of oropharyngeal SCC, a subset of HNSCC (25). The other smaller subsets of SSC arising from the oral cavity, larynx, nasopharynx, and paranasal sinuses may also test HPV-positive (26). Approximately half of the patients diagnosed with SCC originating from an unknown primary site in the head and neck region exhibit HPV-positivity (27). It is well-established that a strong correlation exists between HPV infection and increased risk of oropharyngeal SCC (28, 29). HPV 16 is the most common type in HNSCC, accounting for 90% of the cases, and HPV 18, 33 and 35 are responsible for the small remaining fraction (30).

Numerous studies have consistently shown that HPV-positivity in oropharyngeal SCC is associated with a significantly lower mortality risk and better prognosis compared to HPV-negative cases (31, 32). This may be attributed to the distinct genetic profiles of HPV-negative and HPV-positive HNSCC, which ultimately influence the initiation and progression of the disease (4).

The presence of HPV-mediated disease can be evaluated via direct methods, e.g., the detection of HPV DNA or E6/E7 mRNA detection by either polymerase chain reaction (PCR) or *in situ* hybridization (ISH), or via indirect methods, e.g., the identification of p16 expression via immunohistochemistry (IHC) (33). p16 protein, a CDKN2A gene product, inhibits the cell cycle through binding and inhibiting cyclin-dependent kinase- and 6 (CDK4 and CDK6). When the HPV E7 protein binds phosphorylated retinoblastoma (pRb) protein, it disrupts a feedback mechanism that normally limits p16 expression through free pRb, resulting in p16 overexpression (34).

The impact of HPV status on prognosis in oropharyngeal SCC led to the introduction of distinct staging criteria in the 8th edition of the American Joint Committee on Cancer Manual for HPV-positive and HPV-negative oropharyngeal cancer (35). The most accurate technique for evaluating HPV infection remains the detection of mRNA transcripts. However, a combined strategy involving p16 evaluation by IHC and HPV DNA by PCR has shown similar sensitivity and specificity rates (36). Consequently, the National Comprehensive Cancer Network (NCCN) guidelines recommend the use of p16 IHC as a surrogate biomarker for HPV testing in all patients diagnosed with oropharyngeal SCC. This recommendation stems from the strong agreement observed between p16 expression detected by IHC and HPV status determined by HPV E6/E7 mRNA expression (37–39). However, for non-oropharyngeal cancers, routine HPV testing or p16 testing is not recommended owing to the small proportion and lack of consistent evidence in support of prognostic significance (40).

Recently, assays for circulating tumor HPV DNA (ctHPV DNA) have emerged as promising tools for enhancing post-treatment surveillance. Chera et al., found that detecting ctHPV DNA in two consecutive plasma samples during post-treatment monitoring yielded both 100% positive predictive value (PPV) and negative predictive value (NPV) for identifying biopsy-proven recurrence (41). Similarly, another study reported high per test sensitivity (92.5%) and NPV (99.4%) of ctHPV DNA surveillance in oropharyngeal SCC when matched to physician-reported clinical outcome data, highlighting its potential for early disease recurrence detection (42). Tanaka et al., found that ctHPV DNA polymerase chain reaction had similar NPV (89.7% vs 84.0%) and higher PPV (100% vs 50.0%) compared to positron emission tomography and computed tomography (PET-CT). These findings suggest that post-treatment ctHPV DNA testing complements PET-CT and provides valuable insights for managing patients with HPV-related HNSCC post-therapy (43).

Beyond its role as a prognostic biomarker, HPV status also serves as a predictive biomarker. HPV-positive HNSCC has been observed to exhibit heightened responsiveness to ICI treatment compared to HPV-negative cases because of its distinct intrinsic characteristics and elevated expression of programmed cell death-ligand 1 (PD-L1). This has been supported by the findings of a retrospective study wherein high PD-L1 expression in immune cells (≥5%) provided predictive information in HPV-positive oropharyngeal SCC irrespective of the tumor stage (44). Further, the findings from the HNSCC cohort of the multibasket phase I KEYNOTE-012 trial suggested that HPV-positive patients with HNSCC are more responsive to ICIs, wherein an increased overall response rate (ORR) to pembrolizumab was observed in the former group compared to HPV-negative patients (25% vs 14%) (45). However, the outcomes of phase III KEYNOTE-040 could not corroborate these findings. Further studies investigating other anti-PD-1/PD-L1 agents have yielded mixed results, e.g., increased response rates were observed among HPV-positive patients compared to HPV-negative patients when treated with durvalumab (46), while no differences were seen with atezolizumab (47). These observed disparities could potentially be explained by additional concurrent factors beyond PD-L1 expression and immune infiltration such as smoking (to be discussed further) and tumor mutational burden (TMB), which are believed to influence response to ICIs in HNSCC, although their relevance differs between HPV-positive and HPV-negative disease (48). Therefore, HPV-positivity alone may not serve as a reliable predictive biomarker for response to ICI and should be interpreted in conjunction with other accompanying clinical and molecular biomarkers (49).

#### 2.2.2 Epstein-Barr virus infection status

Recent studies have revealed the presence of EBV-encoded small RNAs (EBERs) in tumor cells from various subsets of HNSCCs, including those originating in the tonsils, hypopharynx, larynx, and tongue (50, 51). The detection of EBER-RNA within carcinoma cells has been linked to a poorer prognosis in these patients (50). Carpen et al., reported that EBER expression to be present in stromal lymphocytes adjacent to the tumor and correlated this finding with HPV-positivity, highlighting a complex interplay between EBV and HPV in these tumors. Patients with EBER-positive but HPV-negative OPSCC were found to have significantly poorer overall survival and disease-free survival than those with HPV-positive OPSCC and slightly worse prognosis compared with the patients with EBER-negative and HPV-negative OPSCC. These findings indicate that EBV infection status may have a prognostic impact among patients with HPV-negative OPSCC (51).

The testing methods for the detection of EBV in tumor include ISH for EBV-encoded RNA and IHC staining for latent membrane protein 1 (LMP1). Comparatively, ISH for EBV-encoded small RNA is generally considered a more sensitive testing method for carcinoma than LMP1 IHC testing. Sensitivity and specificity of ISH have been reported to be 94% and 69%, respectively; whereas for IHC, a sensitivity of 44% and specificity of 93% were found (52). However, the traditional chromogenic ISH for EBERs lacks robustness and sensitivity due to the presence of diffuse, nonspecific signals in the extracellular region. Newer detection methods like RNAscope, PrimeFlow, and ViewRNA use specific probes and amplifiers to enhance signal amplification and analytical sensitivity. However, these advanced techniques are often expensive and time-consuming (53).

#### 2.2.3 Immunoscore

The correlation between elevated levels of tumor-infiltrating lymphocytes (TILs), particularly CD3^+^ and CD8^+^ cells and improved patient outcomes has already been affirmed by various studies (54, 55). Immunoscore, assessing TILs (CD3/CD45RO, CD3/CD8 or CD8/CD45RO) both in the core and invasive margin of tumors, has emerged as a pivotal prognostic marker. It categorizes patients from immunoscore 0 (I0, low densities in both regions) to immunoscore 4 (I4, high densities in both regions) (56). Two real-world investigations assessing the prognostic significance of immunoscore, have shown that patients with an intermediate to high immunoscore (score of 1) generally experience better prognoses compared to those with a low immunoscore (score of 0) (57, 58). Similarly, Wang et al., conducted a study affirming the prognostic significance of immunoscore, reflecting the density of total CD3^+^, CD8^+^, and memory T-cells (CD45RO^+^) within either the tumor or stroma. The findings revealed that patients with a high immunoscore (score of 3-6) exhibited prolonged OS and DFS compared to those with a low immunoscore (score of 0-2) (59). Recently, studies have highlighted that an immunoscore combining the density of CD8^+^, FoxP3^+^, and CD68^+^ cells within stromal and intra-tumoral compartments serves as a significant prognostic marker in HNSCC (60–62). These findings advocate that immunoscore could complement the existing array of biomarkers for predicting patient outcomes in HNSCC.

There is mounting evidence that tumor-infiltrating B cells (TIL-Bs) have a crucial role in tumor regulation; correlating with patient prognosis, immune cell infiltration, and response to immunotherapy (63). Ruffin et al., evaluated patients with HNSCC based on enrichment of B cell signatures in TME and observed that not only higher numbers of TIL-Bs, but also the specific phenotype and localization of TIL-Bs in the TME contribute to overall survival. Additionally, TIL-Bs in HPV-positive and HPV-negative HNSCC exhibit distinct transcriptional profiles. HPV-positive HNSCC patients showed higher levels of TIL-Bs in germinal centers and tertiary lymphoid structures (TLS) with germinal centers, which were found to be associated with better clinical outcomes (64).

#### 2.2.4 Other prognostic biomarkers

In the ever-evolving realm of HNSCC research, continual discoveries of additional prognostic markers under investigation underscore the distinctive nature of the mutational landscape, which is dominated by tumor suppressor genes and activating oncogene mutations. Notably, TP53, CDKN2A, and NOTCH1 emerge as frequently mutated genes in HNSCC, often correlating with poorer OS in patients with HNSCC (65). Elevated levels of telomerase expression, detected in 75-100% of patients, and high telomerase reverse transcriptase (TERT) levels (> 93.8 copies) in cancer tissues have been linked to worse survival compared to cases with low TERT levels (< 93.8 copies; HR, 3.30; 95% CI: 1.98-5.52; p < 0.0001) (66). Tano et al., evaluated the relationship between the expression ratio of mRNAs for the antiapoptotic protein Bcl-2 and the proapoptotic protein Bax (the Bcl-2/Bax ratio) and clinical outcomes in patients with HNSCC; wherein DFS of patients with Bcl-2/Bax ratios ≥ 1.2 was noted to be longer than that of patients with Bcl-2/Bax ratios < 1.2 (67). Eukaryotic translation factor 4E is a protein involved in protein synthesis and its overexpression has been correlated with an increased risk of disease progression and poor prognosis and loco-regional recurrence of HNSCC and hence, it can be an independent prognostic predictor in terms of recurrence and survival in patients with HNSCC (68).

Recently, studies have documented the significance of CXC chemokine receptors (CXCR2, CXCR4, CCR2 and CCR7) in HNSCC tumor progression and organ-specific metastasis. This indicates the role of chemokines and their cognate receptors in HNSCC prognosis (69). Also, the overexpression of cytokeratins, one of the major components of intracellular filament networks found in different tissues, has also been observed to be related to tumor progression and prognosis (70). Melanoma-associated gene (MAGE), with an expression rate of 85.5% to 90% in HNSCC tissue, predicts poor oncologic outcomes like poorer 5-year survival rate, in patients with SCC of the larynx and hypopharynx (71).

Furthermore, the TP53 gene alterations are commonly reported in carcinomas including HNSCC and researchers have found that patients with TP53 wild-type status tend to exhibit higher survival rates compared to those harboring TP53 mutations. Consequently, this genetic marker holds significant potential for both diagnostic and prognostic purposes in HNSCC patients. Siemert et al., determined the potential of pre-therapeutic plasma vascular endothelial growth factor (VEGF) levels as biomarkers for outcomes in HNSCC and observed that patients with plasma VEGF < 26 ng/L had superior nodal, local and loco-regional control leading to significant prolonged progression-free survival (PFS) and event-free survival (72). A study conducted by Stoiber et al., revealed that high expression of β-catenin, a pivotal mediator of white blood cell activation, is linked to better OS, and thus was postulated as a potential marker of better outcomes in patients with HNSCC (73).

Podoplanin is a mucoprotein specifically expressed in the lymphatic endothelium, that influences regulating cell proliferation and lymphatic vascular development. Its overexpression correlates with the histological grade of the tumor and lymph node metastasis, offering prognostic insights (74). Thereafter, a metanalysis involving nine studies delineated that the expression of fibroblast growth factor receptor 1 (FGFR-1) also predicted poor OS (HR, 1.97; 95% CI: 1.49-2.61; p < 0.001) in patients with HNSCC (75).

Circulating tumor DNA is released from apoptotic and necrotic tumor cells. Its tumor-specific characteristics make it a valuable component in liquid biopsy, enabling the detection of minimal residual disease and positioning it as a potential biomarker for HNSCC prognosis and disease monitoring (76). Kogo et al. demonstrated that HNSCC patients who tested negative for ctDNA during follow-up after initial curative treatment of HNSCC had a significantly better prognosis compared to those who tested positive for ctDNA (76). Additionally, another study reported that post-treatment ctDNA was detected in 71.4% of patients with recurrent disease, whereas it was not observed in any of the patients without recurrence or metastasis, indicating its potential to prognosticate early detection of recurrence (77). Additional research is still needed to fully understand the utility of the above-mentioned biomarkers for determining the prognosis of HNSCC.

## 3 Predictive biomarkers for immunotherapy in HNSCC

**Figure 2:**
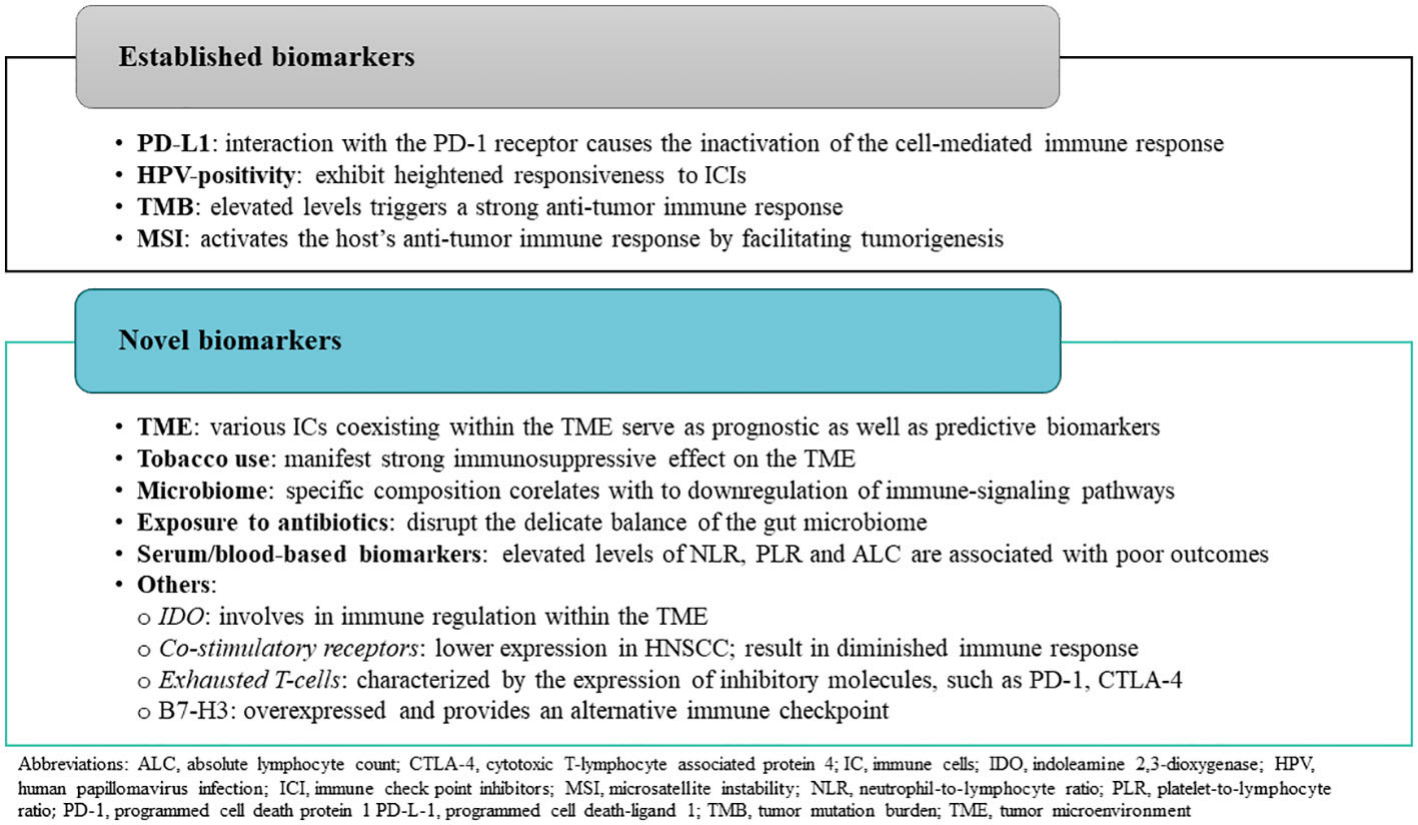
Overview of predictive biomarkers used in head and neck squamous cell carcinoma.

Currently, only next-generation sequencing (NGS) genomic profiling, including testing for combined positive score (CPS), is recommended to be considered to guide patient treatment options, including in clinical trials involving patients with unresectable or metastatic HNSCC. Given the frequent diagnosis of HNSCC at late stages (III/IV) and the significant rates of recurrence, the identification of reliable biomarkers not only for treatment selection but also to evaluate response is essential for optimizing treatment outcomes (73). By utilizing these biomarkers based on the understanding of the tumor biology, therapies can be developed with a very targeted approach allowing the clinicians to maximize treatment efficacy while minimizing the development of adverse effects. Ultimately, the integration of predictive biomarkers into HNSCC management holds the potential to significantly improve patient outcomes by enabling more precise and personalized approaches to treatment. **Figure 2** provides a summary of predictive biomarkers applicable to HNSCC, along with the rationale for their utilization.

### 3.1 Programmed cell death-ligand 1

**Figure 3:**
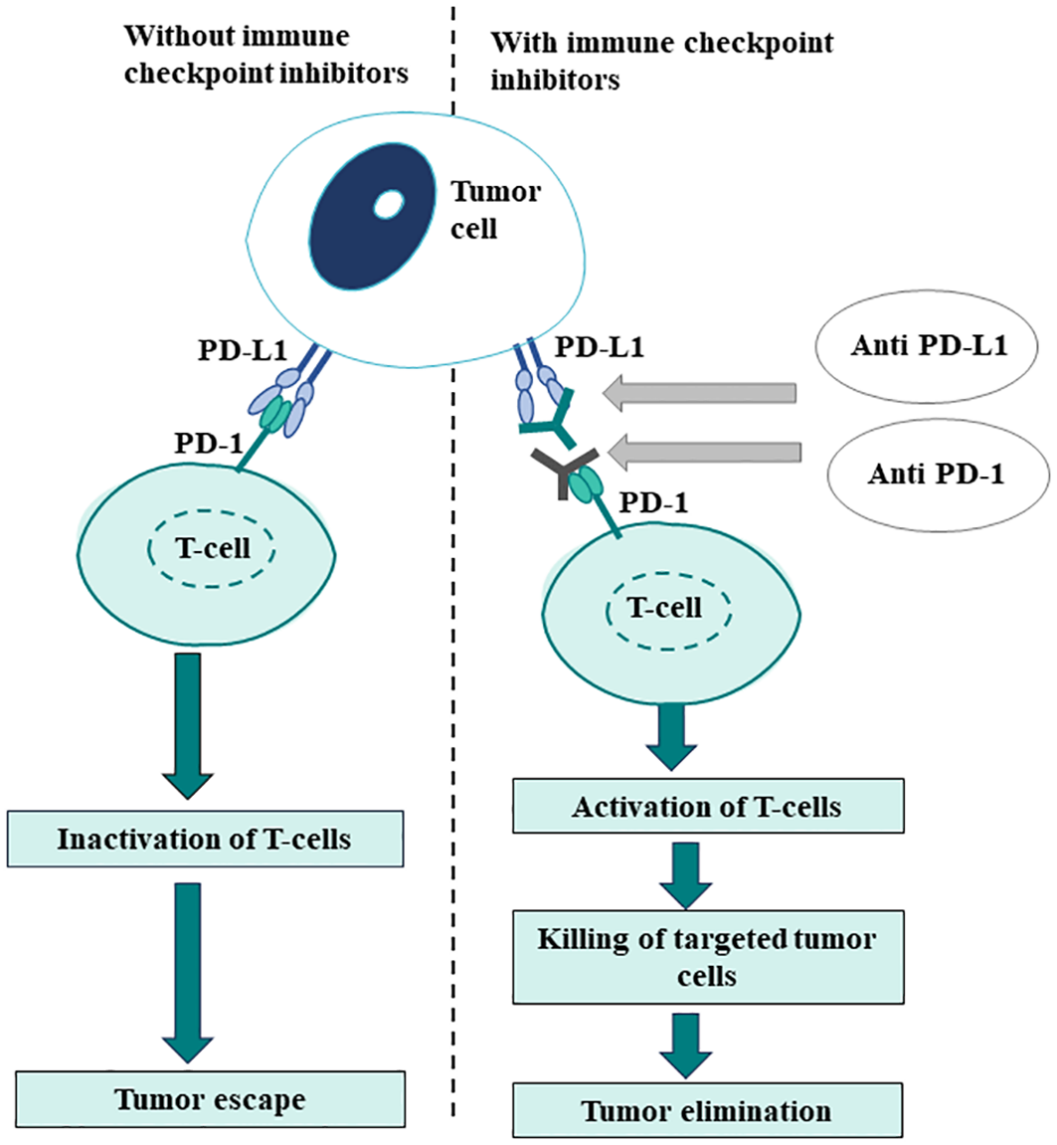
Rationale for PD-L1 expression as a biomarker in immune-oncology.

Programmed cell death-ligand 1, a transmembrane protein, is expressed on tumor cells and tumor-infiltrating immune cells. The interaction of PD-L1 with the PD-1 receptor, expressed on activated T-cells, causes the inactivation of the cell-mediated immune response against tumors (78). Consequently, blocking PD-1/PD-L1 interaction with anti-PD-1/PD-L1 agents facilitates the reactivation of the immune system (**Figure 3**).

The degree of PD-L1 expression within the TME is commonly evaluated by IHC and is represented as either tumor proportion score (TPS) or CPS. TPS measures the percentage of viable neoplastic cells displaying partial or complete membranous staining for PD-L1 at any intensity. CPS calculates the ratio of all PD-L1-expressing cells to the total number of viable tumor cells (79). In the expansion cohort of Phase Ib KEYNOTE-012, CPS was found to be a significant predictor of response in HNSCC. On other hand, TPS, which only considers PD-L1 expression on tumor cells, did not show a significant correlation with treatment response (80). Thereafter, CPS emerged as a preferred scoring system for assessing PD-L1 expression in HNSCC based on the findings of the *post-hoc* analysis of the KEYNOTE-040 study that revealed that the CPS score was more sensitive than the TPS at lower cut-offs (81). This shift requires pathologists to increasingly assess PD-L1 expression and assign CPS scores to aid in treatment selection for HNSCC patients (79).

It has been widely observed that anti-PD-1/PD-L1 agents achieve higher response rates in tumors that express PD-L1 compared to those without PD-L1 expression (11). PD-L1 expression has been found to correlate with clinical outcomes in numerous clinical trials like Keynote 040 and CheckMate 141 involving anti-PD-1/PD-L1 agents. Based on the evidence generated by these clinical studies, anti-PD-1 ICIs nivolumab and pembrolizumab were approved by the US Food and Drug Administration (FDA) for the treatment of patients with recurrent HNSCC that is refractory to platinum-based regimens. Subsequently, following the findings of Keynote 048 trial, pembrolizumab received approval for use in combination with platinum and fluorouracil for all patients with HNSCC, and as a monotherapy for patients whose tumors express PD-L1 CPS ≥ 1 (82).

The correlation between FDA approval for these immunotherapy drugs in HNSCC and PD-L1 expression underscores the pivotal role of PD-L1 as a predictive marker, making it the most widely used biomarker in clinical practice (83). **Table 2** enlists the major clinical studies assessing anti-PD-1/PD-L1 agents tested in recurrent and metastatic HNSCC and showing their response correlation with PD-L1 expression. The discordance of results observed among various studies including anti-PD-1/PD-L1 agents may be due to the lack of uniformity in the assays and the variability in the thresholds used to define PD-L1 positivity. This inconsistency impairs the comparability of results across studies, complicates the interpretation of PD-L1 expression levels, and can affect treatment outcomes by leading to discrepancies in the classification of patients based on PD-L1 status (48). Moreover, PD-L1 expression is intricately regulated by multiple signaling pathways, notably including MAPK, PI3K, and Akt/PKB, which are commonly dysregulated in HNSCC. Consequently, PD-L1 serves as a dynamic biomarker, subject to temporal variations and spatial heterogeneity. Its expression may evolve from the point of initial diagnosis to recurrence or progression, necessitating new biopsy sample (84), and may vary between primary and coexisting metastatic lesions (48, 85). A recent study revealed a relatively low concordance of PD-L1 CPS expression (66.1%) between primary and recurrent tumors, underscoring the necessity of confirmatory biopsies at relapse instead of relying on archival tissue for PD-L1 evaluation (86).

**Table 2:** Immune checkpoint inhibitors investigated for the management of recurrent/metastatic HNSCC.

Drug (target)	Study name (Phase of the study)	Key findings	PD-L1 expression location and cut-off	ORR (%)		OS (HR)^a^	
				PD-L1+	PD-L1-	PD-L1+	PD-L1–
Pembrolizumab (PD-1)	KEYNOTE-012 (Phase I) (45, 80)	• PFS: 23% • OS: 59% • Greater ORR in PD-L1-positive patient versus PD-L1-negative patients (p = 0.021)	TC + IC ≥ 1%	22.00%	4.00%	Not applicable	Not applicable
			TC ≥ 1%	17.00%	7.00%		
	KEYNOTE-040 (Phase III) (139, 140)	• Pembrolizumab vs standard of care OS: 8·4 vs 6.9 months; HR, 0·80, 0·65–0·98; p=0·0161	TC+ IC (CPS ≥ 1)	17.30%	Not available	0.74 (p = 0.0049)	Not available
			TC (TPS ≥ 50)	26.60%	Not available	0.53 (p = 0.0014)	Not available
	KEYNOTE-048 (Phase III) (18, 19)	• Pembrolizumab alone improved OS compared to cetuximab with chemotherapy in patients with CPS ≥20 (14.9 *vs* 10.7 months; p=0·0007) and CPS ≥ 1 (12.3 *vs* 10.3 months; p=0·008) • Pembrolizumab with CT improved OS compared to cetuximab with chemotherapy in the total population (13·0 months *vs* 10·7 months, p=0·0034)	TC + IC (CPS ≥ 1)	19.10%	Not available	0.78 (p *=* 0.0086)	Not available
			TC + IC (CPS ≥ 20)	23.30%	Not available	0.61 (p= 0.0007)	Not available
Nivolumab (PD-1)	CheckMate-141 (Phase III) (141, 142)	• OS was significantly longer with nivolumab than with standard therapy (7.5 vs 5.1 months; HR, 0.70; 97.73% CI, 0.51 to 0.96; p=0.01)	TC ≥ 1%	17.00%	11.80%	0.55	0.73
Durvalumab (PD-L1)	MEDI4736-1108 (Phase II) (143)	• 6 and 12-month OS was 62% (95% CI: 48-74) and 42% (95% CI: 27-55), respectively	TC ≥ 25%	18.00%	8.00%	Not applicable	Not applicable
	HAWK (Phase II) (144)	• Median PFS and OS for treated patients were 2.1 months (95% CI: 1.9-3.7) and 7.1 months (95% CI: 4.9-9.9), respectively	TC ≥ 25%	16.20%	Not available		
	CONDOR (Phase II) (145)	• ORR was 7.8% (95% CI: 3.78%-13.79%) in durvalumab + tremelimumab arm, 9.2% (95% CI: 3.46%-19.02%) for durvalumab monotherapy, and 1.6% (95% CI: 0.04%-8.53%) for tremelimumab monotherapy	TC < 25%	Not available	6.00%		
Atezolizumab (PD-L1)	GO27831 (Phase I) (146)	• ORR: 22%	IC 2/3: > 5%	24.00%	Not available	Not applicable	Not applicable
			IC 0/1: < 5%	14.00%			

CI, confidence interval; CPS, combined positive score; CT, chemotherapy HR, hazard ratio; ICs, immune cells; ORR, overall response rate; OS, overall survival; PFS, progression-free survival; TCs, tumor cells; TPS, tumor proportion score.

a HR for OS resulting from: nivolumab and pembrolizumab versus investigator’s choice of chemotherapy (Docetaxel, Methotrexate and Cetuximab) in the CHECKMATE-141 and KEYNOTE-040 studies, respectively; pembrolizumab monotherapy versus EXTREME regimen in the KEYNOTE-048 study; durvalumab versus tremelimumab plus durvalumab in the CONDOR study.

### 3.2 Tumor mutational burden and neoantigen burden

Tumor mutational burden (TMB) is a measure of the total number of coding mutations in a tumor’s genome and is reported as the number of mutations per megabase (mut/Mb) or muts/exome of DNA sequenced. Elevated TMB has been correlated with increased neoantigens production by tumor cells, triggering a strong anti-tumor immune response, immune evasion by the tumor, and thus correlates with sensitivity to ICIs. The prevalence of TMB-high in head and neck cancers is reported to be in the range of 10% (cut-off of 15 mut/Mb) to 19% (cut-off of 10 mut/Mb) (87, 88). TMB has been reported as a potential biomarker to predict the efficacy of immunotherapy (89). Haddad et al., observed a strong association between TMB and objective response, as well as a positive trend between neoantigen load and clinical response to pembrolizumab in patients with recurrent/metastatic HNSCC (90). Further, a meta-analysis of 1200 patients with HNSCC revealed that patients harboring high TMB exhibited a significantly improved OS rate (OR = 2.62; 95% CI: 1.74–3.94; p < 0.0001) and a survival advantage (HR, 0.53; 95% CI: 0.39-0.71; p < 0.0001) after ICI treatment (91). Similarly, a recent study evaluating 674 patients across 8 cancer types including HNSCC demonstrated that patients with high TMB cancers were significantly associated with longer OS (HR, 0.61; upper confidence bound [UCB], 0.84; p = 0.005), PFS (HR, 0.62; UCB, 0.82; p = 0.003), and time to progression (HR, 0.67; UCB, 0.92; p = 0.02) compared to patients with low TMB cancers. Additionally, patients with high TMB exhibit OS benefits regardless of the type of ICI used (pembrolizumab; HR, 0.67; UCB: 0.94; p = 0.03; other ICIs; HR, 0.37; UCB: 0.85; p = 0.03) (92). To investigate the differences in TME of HNSCC with various PD-L1 expression, a retrospective analysis of 409 patients with HNSCC was performed to compare the immune signatures of tumors with high (CPS ≥ 20), moderate-low (CPS -1 to 19), or negative (CPS <1) PD-L1 expression. The findings of the study revealed tumors with high PD-L1 (CPS ≥ 20) exhibited greater immune infiltration, higher immunogenicity scores, and increased co-inhibitory receptor expression (LAG-3, TIGIT, TIM-3) compared to moderate-low and negative PD-L1 tumors. These findings underscore that understanding the different components of TME can aid in implementing innovative therapeutic strategies to enhance the immune responses in HNSCC (93).

As a result of the emerging evidence, TMB has been gradually gaining prominence as a prospective biomarker for immunotherapy response in patients with HNSCC. However, the presence of numerous subclones of tumor may lead to neoantigen heterogeneity and cause host immune invalidation despite harboring a high TMB. This could be the possible reason for some patients with high TMB not responding to ICI therapy (94). In addition, challenges related to the clinical interpretation of TMB testing results have also been highlighted as currently there are no clear consensus methods for optimal cut-off determination of high and low TMB. A meta-analysis including 11 studies reported a variation in TMB cut-off ranging from 6.0 to 175.0 muts/exome, with a mean of 130.5. Moreover, this study reported variation in the TMB testing methods; most studies used NGS, and a few used whole-exome sequencing (WES). Though WES is the gold standard for measuring TMB, the practical application of WES in clinical settings faces limitations such as high cost, prolonged detection time, intricate data interpretation, and the requirement for fresh samples. On the other hand, the rapid progression of NGS technologies has facilitated expedited genome sequencing, attributed to its exceptional throughput, scalability, and speed. The existing studies have highlighted a significant correlation between TMB measured through WES (95, 96), but uniform industry standards for TMB tested by NGS is the need of the hour.

### 3.3 Microsatellite instability

The mismatch repair pathways play a crucial role in identifying and repairing mismatched bases in DNA replication and genetic recombination, both in normal and tumor cells. DNA mismatch repair involves the mutSα protein complex (responsible for initial recognition of mismatch) made up of heterodimer partners MSH2 and MSH6 and the mutLα protein complex (which initiates DNA repair) made up of heterodimer partners MLH1 and PMS2.^82^ Microsatellite instability (MSI), is a direct consequence of DNA mismatch repair deficiency, characterized by an accumulation of mutations in DNA elements called microsatellites. This accumulation of mutations drives tumorigenesis and also contributes to elevated neoantigen formation and activation of the host’s anti-tumor immune response (97). MSI-high (MSI-H) status results from loss of MSH2, MSH6, MLH1, or PMS2 function through mutation or other mechanisms such as methylation of the MLH1 promoter. MSI-H status can be measured directly by PCR or NGS based techniques and is also strongly correlated with loss of expression of the MMR protein MSH2, MSH6 MLH1, and PMS2 measured by IHC (MMR deficiency). Two studies including 153 and 67 patients with HNSCC respectively, reported the prevalence of MSI-H to be 3% (98, 99). In recent years, it has been demonstrated that MMR deficiency (MMR-D) and MSI, could predict the tumor response to immunotherapies. In two consecutive investigations, T Le et al., found that pembrolizumab elicited an objective radiographic response in 40-53% of patients with MMR-D cancers originated from various organs, in contrast to MMR-proficient malignancies, which did not benefit from the therapy (100, 101). In 2017, the FDA granted accelerated approval to pembrolizumab for treating unresectable solid tumors with MSI-H or MMR-D supported by data from 149 patients across five clinical trials (102). Also, based on the findings of the CheckMate 142 study, nivolumab was approved by the FDA for patients with metastatic colorectal cancer with MSI-H or MMR-D who had disease progression after chemotherapy (103). The findings from the phase II KEYNOTE-158 study revealed that the PD-1 blockade has a promising activity for MSI-H or MMR-D cancer regardless of the tumor type (104). However, there is a segment of the population with MMR-D who failed to effectively respond to the ICI therapy which may be attributed to the lower MSI intensity or to the alterations in tumor antigen-presenting machinery and tumor-extrinsic factors such as inadequate T-cell activation that have an impact on the response to ICIs (105). For detecting MSI, PCR testing is regarded as the gold standard, but the test is costly and requires control testing of non-malignant tissues. The alternative, IHC is cheaper and clinically more accessible. It is recommended to perform IHC staining for expression of all four MMR proteins: MLH1/PMS2 and MSH2/MSH6 to get both qualitative and quantitative results (106).

## 4 Novel biomarkers

### 4.1 Tumor-infiltrating cells and tumor microenvironment

The growth of a tumor is determined by the dynamic interaction between the tumor and its microenvironment. The composition of immune cells within the TME, referred to as immune contexture, not only carries prognostic significance but also serves as a predictive factor for responses to immunotherapies (107). Mandal et al., observed an increased density of immune-infiltrating cells, specifically CD56^+^ natural killer (NK) cells, and found it to be correlated with a better ORR in patients with HNSCC treated with different ICIs (108). Similarly, Hanna et al., reported that patients with HNSCC with a high CD8^+^ lymphocyte rate and PD-1 expression were correlated with improved response rates with ICIs (109). Tumor-infiltrating cells can be evaluated in routine HE-stained slides of different subsites of HNSCC. The integration of digital analysis tools with HE-stained slides further enhances the accuracy, efficiency and reproducibility of the evaluation process (110). The accumulating evidence suggests that these tumor-infiltrating cells may provide a useful, additional parameter to assess tumor immune response, which requires further examination in prospective studies using standardized methodology (110).

### 4.2 Tobacco use

Tobacco use has a strong immunosuppressive effect on the TME in patients with HNSCC and therefore also seems to influence the response to checkpoint inhibition in patients with HNSCC. Within the CheckMate-141 study, subgroup analysis indicated a trend toward reduced survival benefits from nivolumab among smokers compared to nonsmokers (20). Similarly, a retrospective analysis of 81 HNSCC patients revealed that former/current smokers were less responsive to anti-PD-1/PD-L1 agents compared to patients who never smoked. However, this correlation remained significant only among HPV-negative patients, suggesting the immunosuppressive effects of smoking may not be as significant in HPV-positive tumors (111).

### 4.3 Oral microbiome

Mucous membranes in the oral cavity and pharynx, are continually exposed to environmental factors capable of modifying the oral microbiota. Pushalkar et al., discovered distinct oral microbiota composition in patients with oral SCC compared with healthy controls (112). Similarly, Prestin and colleagues noted a substantial reduction in the richness and diversity of microbiota species in patients with HNSCC compared to the controls. Additionally, they observed differential microbiota enrichment in HPV-positive and HPV-negative oropharyngeal and oral SCC, indicating the specific microbiota presence according to tumor location and HPV status. The authors also found a decrease in the alpha diversity post-surgery, with an increase in patients experiencing recurrence (113). In alignment with these findings, another study examining the oral microbiota present in the saliva of HNSCC patients before and after treatment (including surgery, chemoradiotherapy and ICI) revealed associations between specific oral bacteria composition (*Fusobacterium* and *Lactobacillus*), downregulation of immune-signaling pathways and upregulation of oncogenic Wnt/Beta-catenin pathways (involved in carcinogenesis and development of HNSCC) (114). Collectively, these findings suggest that the oral microbiota holds promise as a potential prognostic and predictive biomarker for HNSCC.

### 4.4 Exposure to antibiotics

Various studies have indicated a negative impact of antibiotic exposure on immunotherapy outcomes. Wada et al., reported that the use of proton-pump inhibitors (PPIs) and antibiotics independently attenuated the efficacy of nivolumab (OS; PPIs: HR, 1.70; 95% CI: 1.01-2.87, p = 0.046; antibiotics: HR, 1.85; 95% CI: 1.00-3.41, p = 0.048) in recurrent/metastatic HNSCC (115). In a systematic review, both OS (HR, 2.07; 95% CI: 1.51-2.84; p < 0.01) and PFS (HR,1.53; 95% CI: 1.22-1.93; p < 0.01) was found be inferior in patients using antibiotics (116). Similarly, a retrospective study involving a large cohort of patients reported that any antibiotic exposure within 1 year before ICI was associated with worse OS (HR, 1.12; 95% CI: 1.12-1.23; p = 0.03). Notably, fluoroquinolones were found to have the strongest impact on OS, likely due to their ability to alter many gut microbiota species crucial for ICI responses such as *Faecalibacterium*, *Ruminococcus*, *Bifidobacteria*, and *Alistipes* (117). The growing evidence highlights the predictive significance of antibiotics in ICI efficacy. It also emphasizes the need to carefully assess and reduce antibiotic usage in patients receiving ICI treatment, to preserve a balanced gut microbiome and optimize therapeutic outcomes of immunotherapy.

### 4.5 Serum/blood-based biomarkers

The neutrophil-to-lymphocyte ratio (NLR) is a widely researched parameter in peripheral blood analysis that reflects the level of specific immunity. In a meta-analysis involving 6479 patients with HNSCC, elevated NLR before treatment was found to be associated with shorter OS. The study included various HNSCC subgroups such as the oral cavity, nasopharynx, hypopharynx, and larynx. The overall HR for OS was determined to be 1.78 (95% CI: 1.53-2.07), indicating that patients with elevated NLR before treatment had a poorer prognosis (118). Similarly, another meta-analysis of 929 patients with HNSCC reported higher NLR to be associated with poor OS (HR, 2.03; 95% CI: 1.50-2.74), PFS (HR; 2.15, 95% CI: 1.44-3.21), and disease control (OR, 0.30; 95% CI: 0.12-0.74) (119). Park and colleagues observed that both low NLR (<6.2) and high absolute lymphocyte count (≥0.77) at week 6 were associated with a longer PFS (5.6 vs 3.1 months, p = 0.002; and 8.7 vs 2.9 months, p = 0.001, respectively) in patients receiving ICIs for HNSCC management (120). Further, a meta-analysis of 13 studies showed an elevated platelet-to-lymphocyte ratio to be significantly associated with poorer OS (HR, 1.85; 95% CI: 1.35-2.52; p < 0.00001) and disease-specific survival (HR, 1.57; 95% CI: 1.25-1.97; p < 0.0001) (121). A recently conducted study hypothesized that there might be associations between the frequencies of human leukocyte antigen (HLA) alleles and the survival of tumor patients, and the findings revealed that HLA-A*02, the most prevalent allele in patients with HNSCC, was associated with improved OS and PFS (HR, 0.54; 95% CI: 0.31-0.92; p = 0.023). HLA-A*02 allele expression might not only predict better survival but also indicate enhanced tumor antigen presentation, potentially aiding in selecting patients with HNSCC for T-cell-dependent immunotherapies (122).

### 4.6 Others

#### 4.6.1 Copy number alterations

Copy number alterations (CNAs) have been frequently observed in HNSCCs, for instance, deletion of the tumor suppressor gene CDKN2A in 9p21.3, which is the most prevalent gene‐specific CNA, has been found in 30.9% of HNSCCs. Other common cancer gene‐specific CNAs in HNSCC are amplifications of CCND1 and FGF3/4 in 11q13.3 (23.2%), PIC3CA in 3q26.32 (15.7%), TP63 in 3q28 (16.1%), EGFR in 7p11.2 (10.4%), and MYC in 8q24.21 (9.3%). CNAs have been reported as an independent prognosticate of worse prognosis in patients with recurrent/metastatic HNSCC receiving immunotherapies (123).

#### 4.6.2 Interferon-γ and other gene expression profiles

Ayers et al., demonstrated that an IFN-γ–related gene profile obtained from baseline tumor tissue was predictive of the best overall response and PFS in cohorts of patients with melanoma, HNSCC, and gastric cancer who were treated with pembrolizumab (124). Further, a retrospective study identified five most significantly overexpressed immune-related genes in HNSCC: TNFRSF9/4-1BB (77%), IDO1 (75%), TNFSF4/OX40L (74%) and TNFRSF18/GITR (74%), and FOXP3 (62%). The majority of the analyzed tumors overexpressed actionable immunity genes, including PD-1/PD-L1, TIGIT, OX40/OX40L and/or cytotoxic T-lymphocyte associated protein-4 (CTLA-4). The overexpression of these genes was found to be directly related to clinical outcomes. Haddad et al., found 18-gene T-cell-inflamed gene expression profile (GEP) to be associated with PD-L1, indicating that GEP may prove helpful in predicting response to anti-PD-1/PD-L1 agents in HNSCC (90).

#### 4.6.3 Cancer-associated fibroblasts

The functional significance of distinct subsets of head and neck cancer-associated fibroblasts (HNCAFs) in modulating the immunoregulatory environment of HNSCC has been highlighted by a few researchers. Specifically, HNCAF-0/3 was identified as predictive of nivolumab response, whereas HNCAF-1 was associated with immunosuppression (125).

#### 4.6.4 Growth factors

Epidermal growth factor receptor is overexpressed in more than 90% of HNSCC cases and its overexpression corresponds to tumor growth and progression, resistance to therapy and poor outcomes for patients with HNSCC (126). Cetuximab, a monoclonal antibody targeting the extracellular part of EGFR, has shown improvement in survival rates when combined with radiation compared with radiation alone among patients with HNSCC.

#### 4.6.5 Tumor-derived exosomes

Recent research indicates that measuring total exosome levels, the ratio of tumor-derived exosomes (TEX) and total exosome and the specific characteristics of exosomes originating from TEX or T-cells could effectively differentiate between patients with HNSCC who positively responded to tumor treatment and those who did not (127).

#### 4.6.6 Indoleamine 2,3‐dioxygenase

A systematic review assessed 40 clinical studies to elucidate the role of indoleamine 2,3‐dioxygenase (IDO), a key intracellular enzyme involved in immune regulation, in HNSCC and found that its immunohistochemical expression was correlated with worse survival. Moreover, IDO expression was found to be correlated with positive PD‐L1 and HPV status and therefore it was proposed as an emerging predictive biomarker for response to PD‐L1 therapy (128).

#### 4.6.7 Co-stimulatory receptors

Patients with HNSCC have shown lower expression of co-stimulatory receptors, resulting in T-cell anergy/apoptosis and diminished immune response (129). GSK609, an agonist of inducible T-cell co-stimulatory, has been investigated in combination with pembrolizumab, in patients with HNSCC who have failed platinum-based therapy. The drug combination has shown encouraging outcomes (ORR, 26%; 95% CI: 12.9-44.4); disease control rate, (68%; 95% CI: 49.5-82.6) suggesting that this combination therapy can effectively activate T-cells and overcome the immune suppression observed in HNSCC (130).

#### 4.6.8 B7-H3

B7-H3 (also known as CD276) is a member of the B7 ligand family, overexpressed in HNSCC, and provides an alternative immune checkpoint to therapeutically target alone or in combination with PD-1-targeted therapies. Recently, in a phase I/II trial, enoblituzumab (anti-B7-H3 antibody) combined with pembrolizumab in post-platinum PD-L1-naive recurrent/metastatic HNSCC showed 33% of patients to experience ORR (131).

#### 4.6.9 Exhausted T-cells

The exhausted T-cells are characterized by the expression of inhibitory molecules, such as PD-1, CTLA-4, T-cell immunoglobulin, and immunoreceptor tyrosine-based inhibitory motif: lymphocyte activation gene 3 (LAG-3), T-cell immunoglobulin and mucin domain (TIM-3) and others, that negatively regulate their response, as well as reduced secretion of cytokines and cytolytic molecules (132). The expression of TIM-3, and LAG-3 have been observed to be overexpressed in HNSCC and are reported to be associated with poor prognosis (133). A phase II neoadjuvant study to determine the safety and tolerability of nivolumab alone or in combination with anti-LAG-3 (relatlimab) or anti-CTLA-4 (ipilimumab) in resectable HNSCC is under clinical trial (134).

#### 4.6.10 Transforming growth factor

The defective transforming growth factor (TGF) signaling in HNSCC results in cancer invasion and metastasis and helps tumor cells escape from immune surveillance. A notable increase in TGF levels has been observed in patients with HNSCC patients (135), suggesting its potential as both a marker for malignant transformation and a target for preventive therapy.

#### 4.6.11 Enoblituzumab

The efficacy of enoblituzumab in combination with retifanlimab (anti-PD-1) or tebotelimab (engineered against PD-1) for the treatment of patients with recurrent/metastatic HNSCC is currently under investigation.

Apart from identifying a reliable biomarker associated with response to a given ICI, the forthcoming wave of novel immunotherapeutic approaches combining different ICI with co-stimulatory agonists, therapeutic vaccines and cytotoxic drugs, has recently led to the concept of targeting ‘hot’ tumors. Hot tumors are characterized by an overall activated immune microenvironment with high infiltration of immune cells and are more likely to respond to immunomodulatory therapies. Using gene expression profiles of 421 HNSCC, Foy and colleagues developed and validated a score to identify immunologically active tumors. The 27-gene expression-based ‘HOT’ score was observed to be correlated with PD-L1 and IDO expression CD8 infiltrate and activation of the IFN-γ pathway. Additionally, among patients with HNSCC treated with ICIs, the HOT score was linked with an improved OS and PFS, indicating that this simple and robust approach can be utilized to identify real-world patients with immunologically active HNSCC who may benefit from ICIs (136). Additionally, leveraging specialized tissue models that closely mimic the native TME, encompassing immune signatures and intratumor heterogeneity, enhances the precision of testing targeted therapies. One such innovative tissue model with transformative potential in cancer research and treatment is the organoid model. Organoids are 3D, multicellular *in vitro* tissue constructs that mimic specific *in vivo* organs and can be utilized to explore physiological and pathological aspects associated with that organ. In the case of HNSCC, organoids offer the potential to uncover novel subsets of immune markers for targeted therapy, given that drug screenings have demonstrated sensitivity to targeted drugs not traditionally employed in HNSCC treatment (11), thereby paving the way for expanded therapeutic avenues.

## 5 Challenges and future perspectives

In recent decades, biomarker discovery has seen remarkable growth, largely driven by advances in high-throughput technologies. However, validating these biomarkers in large patient cohorts remains challenging due to the high costs of validation and the absence of objective prioritization criteria. Reliable biomarker testing requires high-quality tissue preparation, which depends on well-preserved and properly processed samples, necessitating precise coordination between biopsy procedures and laboratory protocols. The substantial expense of advanced genomic testing further complicates integration, creating financial barriers for the patients. Accessibility remains an issue, as these advanced tests are not consistently available across all healthcare settings, particularly in rural or underserved areas. Additionally, implementing these biomarkers requires specialized infrastructure, including advanced analytical equipment and skilled personnel. Addressing these challenges demands improving sample handling, managing costs effectively, increasing test accessibility, and making significant investments in technology and professional training (137).

The present review article provides a valuable synthesis of current knowledge and advancements in the field of HNSCC. Its focused examination on established and emerging biomarkers and their role in tailoring immunotherapy for HNSCC, may potentially guide future research and clinical practices. However, the limited number of patients included in the reviewed studies may affect the generalizability of the findings, and variations in research standards across studies pose challenges for comparing results.

Future challenges in the field include leveraging multi-omic profiling to better understand biomarker heterogeneity, advancing research to uncover and target resistance mechanisms through innovative therapies, and optimizing treatment sequencing by integrating patient-specific data and predictive models to enhance personalized treatment approaches.

## 6 Conclusion

The evolving landscape of HNSCC underscores the crucial need for early detection and appropriate prognosis. Despite numerous exploratory investigations, translating prognostic and predictive biomarkers findings into clinical practice is hindered by the lack of prospective clinical trials and validation studies.

Immunotherapy is a promising approach for HNSCC treatment. However, understanding patient response to immunotherapy is multifaceted and is influenced by various factors. This underscores the necessity for robust and clinically applicable predictive biomarkers that can reliably guide patient selection and treatment decisions. Histopathological analyses are crucial for understanding tumor characteristics and guiding treatment. Integrating established and novel biomarkers into this framework enhances treatment precision and personalization. These advancements hold the potential for improving outcomes and quality of life for those affected by these diverse and challenging diseases. Collaborative efforts of researchers, clinicians, and pharmaceutical industries are indispensable for identifying and validating these biomarkers, aiming to optimize patient outcomes through personalized treatment strategies.

Advancements in molecular testing techniques like NGS are crucial for assessing the current and emerging biomarkers and tailoring therapies. Centralization of predictive molecular testing within specialized laboratories, integrated into regional oncology networks, is essential to ensure equitable access and cost-effectiveness.
